# Editorial: AMPK and mTOR Beyond Signaling: Emerging Roles in Transcriptional Regulation

**DOI:** 10.3389/fcell.2020.641552

**Published:** 2021-01-15

**Authors:** Étienne Audet-Walsh, Mathieu Vernier, Benoit Viollet

**Affiliations:** ^1^Department of Molecular Medicine, Faculty of Medicine, and Endocrinology—Nephrology Research Axis, Centre de Recherche du CHU de Québec, Université Laval, Québec City, QC, Canada; ^2^McGill University and the Goodman Cancer Research Centre, Montréal, QC, Canada; ^3^Institut Cochin, Université de Paris, CNRS, INSERM, Paris, France

**Keywords:** AMPK, mTOR, metformin, cell metabolism, nucleus, transcriptional regulation, rapamycin, post-translational modifications

Unraveling the signal transduction processes orchestrated by AMPK and mTOR in health and disease has attracted an intense and continual interest in the recent decades. Indeed, mTOR actively promotes growth and proliferation when nutrients are abundant and in response to growth factors. Conversely, when nutrients become limited or under energetic stress, AMPK is activated and inhibits mTOR until a homeostatic energetic state is restored. Encouraged by the development of pharmacological AMPK and mTOR modulators, recent studies have paved the way toward promising clinical applications. However, much remains to be understood regarding the plethora of pathways regulated at the transcription level by AMPK and mTOR as well as all the transcription factors and other mechanisms involved in such regulation. Interestingly, both AMPK and mTOR have been shown under specific cellular cues to: (1) relocalize to the nucleus; (2) be recruited to the chromatin; (3) alters histone marks; and (4) directly or indirectly phosphorylates several transcription factors (Figure 1) (Bungard et al., 2010; Audet-Walsh et al., 2017, 2018; Khan and Frigo, 2017; Giguere, 2020). The objective of the current Research Topic was to further highlight the emerging roles of AMPK and mTOR signaling pathways in the regulation of transcription. The Topic includes four reviews and three original research articles that, collectively, reflect the tremendous importance of transcriptional regulation by AMPK and mTOR signaling pathways contributing to the cellular adaptive response to various metabolic and oxidative stress through all lifespan, from reproduction to cell development and differentiation.

**Figure 1:**
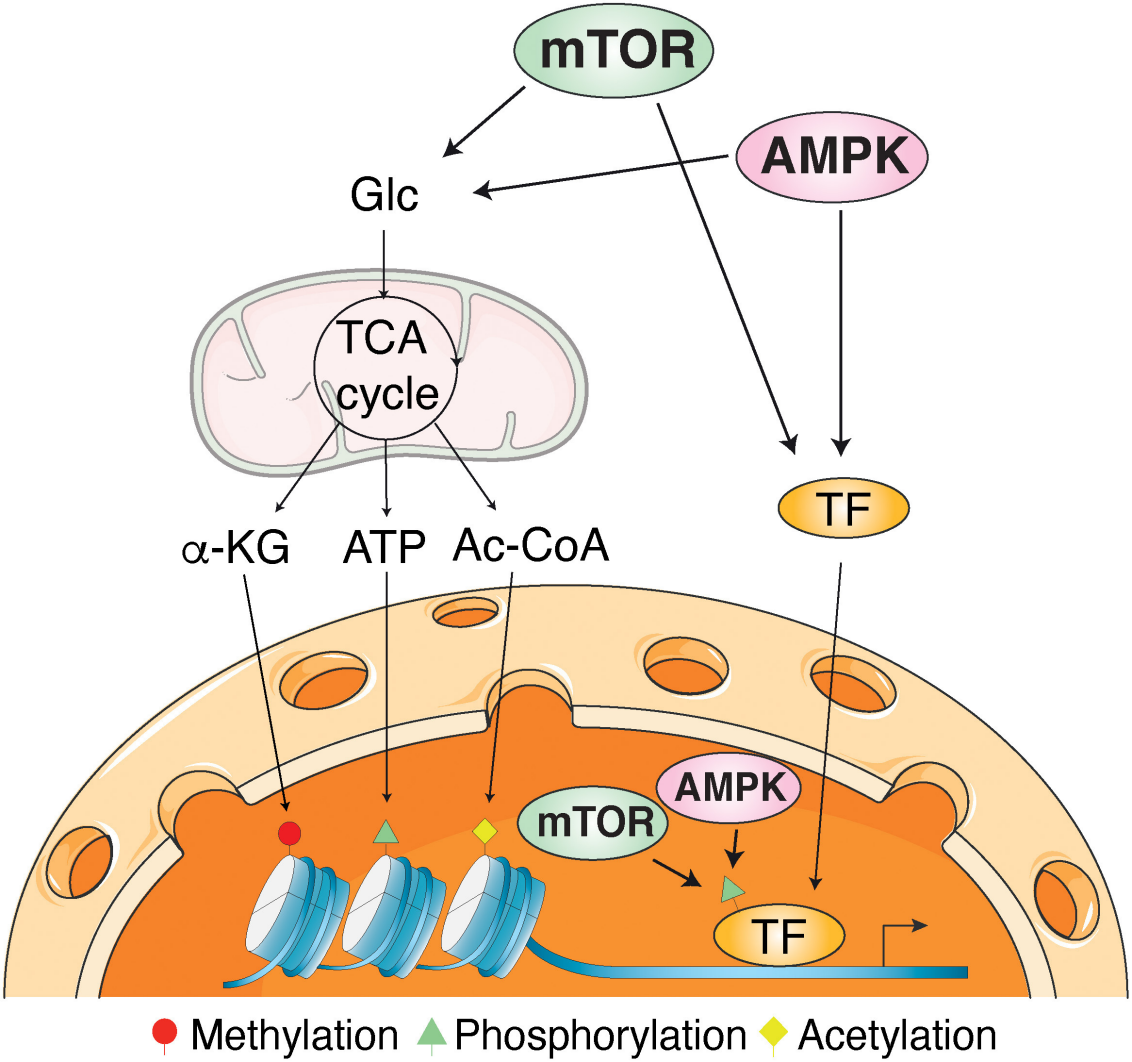
Mechanisms of transcriptional regulation by AMPK and mTOR signaling. AMPK and mTOR influence transcriptional regulation through post-translational modifications of transcription factors and histones as well as epigenetic regulation through the modulation of metabolites produced by intermediary metabolism. Protein post-translational modifications requires ATP for phosphorylation, acetyl-coenzyme A (Ac-CoA) for acetylation, and alpha-ketoglutarate (α-KG) for DNA and histone demethylation. Glc, glucose; TCA cycle, tricarboxylic acid cycle.

The AMPK and mTOR signaling pathways are emerging as major players that integrate several cellular processes in the reproductive system and embryonic development. In a comprehensive overview, Yang et al. detailed the role of AMPK signaling in the control of reproduction as well as in reproductive system related diseases. While mounting evidence supports the role of AMPK in the control of the reproductive neuroendocrine axis, the influence of AMPK on transcriptional events in male spermatogenesis and female fertility remains poorly explored. The authors summarized the potential impact of AMPK on transcriptional regulation in the context of embryonic development, spermatocyte DNA damage repair and gonadal steroid hormone production. Complementary, the article from Wu et al. summarizes the recent evidences linking AMPK to the regulation of milk production in mammals. Following pregnancy, lactation, a phenomenon unique to mammals, represents a major energy investment that has to be tightly regulated. The authors describe the different mechanisms that activate AMPK in the mammary gland, from negative energy balance to heat stress. They then describe how AMPK activation promotes the synthesis of the different milk components by a coordinated regulation of enzymes and transcription factors.

Next, an original article from He et al. demonstrates the involvement of AMPK in the G2 arrest of oxidatively damaged mouse zygote (He et al.), the cell in eukaryotes that is created following egg fecundation by a spermatozoid. Here, the authors shed light on the existence of a protective role of AMPK when zygotes are exposed to mild oxidative stress, by activating the p53/p21 axis to trigger G2 arrest and promote DNA repair. A few days after fecundation, a layer of trophoblasts surrounds the developing embryo and will contribute to the formation of the placenta. Rosario et al. investigated the regulation of the trophoblast transcriptome by the mTOR signaling pathway. To model decreased placental mTOR complex 1 (mTORC1) signaling in human placentas collected from pregnancies complicated by intrauterine growth restriction, the authors silenced raptor, a key mTORC1 regulator, in primary human trophoblast cells. Analysis of global gene changes showed that mTORC1 signaling controls regulatory networks of genes involved in ribosome and protein synthesis, mitochondrial function, lipid metabolism, nutrient transport, and angiogenesis. These studies further confirm the vital importance of transcriptional regulation by AMPK and mTOR in the very first stages of development.

The research article by Fischhuber et al. then describes the discovery of a functional link between AMPK and the transcription factor nuclear factor erythroid 2-related factor 2 (Nrf2) (Fischhuber et al.). They showed, using pharmacological and genetic approaches, that AMPK is essential for the maximal transcriptional regulation of specific genes by Nrf2. Pathway enrichment analysis revealed that these genes are mostly enriched in the PI3K/Akt signaling pathway, an upstream regulator of mTOR. Mechanistically, the authors showed that AMPK inhibited the binding of BTB and CNC homology 1 (Bach1) to the antioxidant response element (ARE). Bach1 being a transcriptional repressor, its inhibition favored Nrf2 recruitment to ARE and the induction of these genes. In a review focusing on the involvement of mTOR in transcriptional regulation of natural killer (NK) cell development, Yang and Malarkannan illustrate the central role of both mTORC1 and mTORC2 in NK cells differentiation and maturation. Through a complete overview, the authors first summarize the importance of mTOR activation by the IL-2/IL-15R cytokine receptors to commit lymphoid progenitors to the NK lineage. Then, based on the most recent transcriptional profiling of NK cells performed by single-cell RNA-sequencing, they detail the different downstream transcriptional mechanisms triggered by mTORC1 or mTORC2 to, respectively, promote early developmental stages and maturation of NK cells, a key component of the innate immune system.

Finally, with a review describing the transcriptional regulation of AMPK and mTOR pathway genes and how these signaling pathways cause durable phenotypic changes through modulation of gene expression, Sukumaran et al. provided a valuable general overview showing the conservation of these regulatory functions from yeast to mammals. The authors highlighted the crucial role of post-translational modifications, mainly direct phosphorylation, of transcription factors and histones as well as epigenetic regulation through the modulation of metabolites produced by intermediary metabolism (Figure 1). They also presented a computational analysis revealing the identification of common transcription factors regulating most of the AMPK and mTOR pathway genes, suggesting a coordinated transcriptional regulation mechanism to maintain abundance and stoichiometry in response to various environmental cues.

## Concluding Remark

It is clear that AMPK and mTOR signaling integrates multiple molecular pathways. In that context, decoding their impact on transcriptional regulation network under physiological and pathological conditions is a significant future challenge to identify novel biomarkers and therapeutic targets.

## Author Contributions

All authors listed have made a substantial, direct and intellectual contribution to the work, and approved it for publication.

## Conflict of Interest

The authors declare that the research was conducted in the absence of any commercial or financial relationships that could be construed as a potential conflict of interest.
